# Phosphate stable oxygen isotope variability within a temperate agricultural soil

**DOI:** 10.1016/j.geoderma.2016.09.020

**Published:** 2017-01-01

**Authors:** Steven J. Granger, Paul Harris, Sabine Peukert, Rongrong Guo, Federica Tamburini, Martin S.A. Blackwell, Nicholas J.K. Howden, Steve McGrath

**Affiliations:** a Rothamsted Research, North Wyke, Okehampton, Devon EX20 2SB, United Kingdom; b Queen's School of Engineering, University of Bristol, Senate House, Tyndall Avenue, Bristol BS8 1TH, United Kingdom; c Institute of Agricultural Sciences, ETH Zurich, Research Station Eschikon 33, 8315 Lindau, Switzerland; d Rothamsted Research, West Common, Harpenden, Hertfordshire, AL5 2JQ, United Kingdom

**Keywords:** Phosphorus, Grassland, Spatial analysis, GW model, North Wyke farm platform

## Abstract

In this study, we conduct a spatial analysis of soil total phosphorus (TP), acid extractable phosphate (PO_4_) and the stable oxygen (O) isotope ratio within the PO_4_ molecule (δ^18^O_PO_4__) from an intensively managed agricultural grassland site. Total P in the soil was found to range from 736 to 1952 mg P kg^− 1^, of which between 12 and 48% was extractable using a 1 M HCl (HCl_PO_4__) solution with the two variables exhibiting a strong positive correlation. The δ^18^O_PO_4__ of the extracted PO_4_ ranged from 17.0 to 21.6‰ with a mean of 18.8‰ (± 0.8). While the spatial variability of Total P has been researched at various scales, this is the first study to assess the variability of soil δ^18^O_PO_4__ at a field-scale resolution. We investigate whether or not δ^18^O_PO_4__ variability has any significant relationship with: (i) itself with respect to spatial autocorrelation effects; and (ii) HCl_PO_4__, elevation and slope - both globally and locally. Results indicate that δ^18^O_PO_4__ was not spatially autocorrelated; and that δ^18^O_PO_4__ was only weakly related to HCl_PO_4__, elevation and slope, when considering the study field as a whole. Interestingly, the latter relationships appear to vary in strength locally. In particular, the δ^18^O_PO_4__ to HCl_PO_4__ relationship may depend on the underlying soil class and/or on different field managements that had operated across an historical north-south field division of the study field, a division that had been removed four years prior to this study.

## Highlights

• We examine the variability of soil P and its δ^18^O_PO_4__ within a 7.1 ha field.
• The acid extractable δ^18^O_PO_4__ was found to range between 17.0 and 21.6‰.
• The δ^18^O_PO_4__ was not spatially autocorrelated and was randomly distributed.
• Variation in δ^18^O_PO_4__ appears heterogenically related to HCl_PO_4__.
• This relationship may depend on historical management of the study field.

## 1 Introduction

Phosphorus (P) is an essential element for plant growth and is applied to agricultural systems, often in large quantities, to underpin intensive levels of agricultural production (Haygarth et al., 2014). It can be applied as either inorganic mineral fertilizers, or via the spreading of animal wastes or other organic materials. Such wastes can occur either directly voided by the animal in the field or applied in bulk after storage while animals are housed. However, P can have significant detrimental effects when they move from land into water bodies. These effects range from direct toxicity (Lewis and Morris, 1986) through to indirect consequences such as eutrophication (Smith et al., 1999). Surface waters are particularly sensitive to P because critical concentrations of only a few tens of μg of phosphate (PO_4_) can cause eutrophication, but are an order of magnitude lower than soil PO_4_ concentrations required for crop growth (Heathwaite and Dils, 2000). Identifying the different pollutant sources that are impacting on a water body is critical to understand its ecosystem health. However apportioning a pollutant to any given source or sources is fraught with difficulty and in recent years techniques have been developed to try to elucidate pollutant origins (e.g. Baker, 2002, Collins et al., 1997, Old et al., 2012) and these include the use of natural abundance stable isotope ratios (e.g. Amberger and Schmidt, 1987, Granger et al., 2008). More recently still the stable isotope approach has been applied to P and although P only has one stable isotope, the technique uses the stable oxygen (O) isotope ratio within the PO_4_ molecule (δ^18^O_PO_4__) to isotopically characterise PO_4_ sources and transformations. However, data on the δ^18^O_PO_4__ of different PO_4_ sources remains limited (Tamburini et al., 2014, Young et al., 2009).

One potential source of PO_4_ in water is from soil, which often receives P inputs in excess of requirements resulting in P accumulation within the soil (Haygarth et al., 1998a). Therefore, as with many other soil properties, an understanding of soil P variability is essential for designing sampling strategies or the evaluation of the effectiveness of diffuse water pollution mitigation measures (Goovaerts, 1998, Rivero et al., 2007). Despite this, few studies describe spatial variability of soil properties and their inter-relationships at a landscape scale (i.e. Marriott et al., 1997, Page et al., 2005). The spatial variability of a given soil property may be related to the combined action of several physical, chemical or biological processes that act at different spatial scales depending on the soil property and process of interest (Goovaerts, 1998). Soil spatial variability is present in both natural and agricultural systems, even if the latter have a long-term uniform management history (Goovaerts, 1998, Marriott et al., 1997). Understanding soil spatial variability is essential to land-based experiments at all scales and its omission is detrimental to the conclusions drawn from such experimental data.

In this study, we conduct a spatial analysis of soil δ^18^O_PO_4__ from an intensively managed agricultural grassland site. While the spatial variability of total P has been described at different scales by other researchers (e.g. Glendell et al., 2014, Page et al., 2005, Penn et al., 2007), this is the first study to assess the variability of soil δ^18^O_PO_4__ at a field-scale. We investigate whether or not δ^18^O_PO_4__ variability has any significant relationship with: (i) itself with respect to spatial autocorrelation effects; and (ii) soil P (extracted using 1 M HCl (HCl_PO_4__)), elevation and slope - both globally and locally within the study field. In particular, the following four null hypotheses are tested:

A. δ^18^O_PO_4__ does not strongly co-vary with HCl_PO_4__, with elevation and with slope across the field as a whole.
B. δ^18^O_PO_4__ is not a spatially-autocorrelated process.
C. The relationships of (A) do not significantly change in different areas of the field.
D. The relationship between δ^18^O_PO_4__ and HCl_PO_4__ is not conditional on the the under-lying soil class or different management histories.

Furthermore, in order to efficiently test the given set of null hypotheses, all statistical analyses are conducted in a manner that accounts for a certain sub-optimality in the study sample design. In particular, statistical methods are chosen to cater for significant areas of under- and over-sampling.

## 2 Methods

### 2.1 Study site

To characterise soil P spatial variability, a series of soil samples were collected from one field of the Rothamsted Research ‘North Wyke Farm Platform’, in south-west England (50.8°N, 3.0°W). The field sampled, referred to as ‘Great Field’, was located on a north-west facing hillslope and comprises clay loam soil overlying the shales and thin subsidiary sandstone bands of the Crackington formation (Harrod and Hogan, 2008). The soils can be further divided in three main types; Hallsworth (USDA Aaerichaplaquept, FAO Stagni-verticcambisol), Halstow (USDA typichaplaquepts, FAO dysticgleysol), and Denbigh (USDA Dysticeutrochrept, FAO Stagni-eutriccambisol) (Harrod and Hogan, 2008). The long-term annual temperature and rainfall are 9.6 °C and 1056 mm, respectively, with a high proportion of rainfall occurring between October and March resulting in waterlogged soils.

Records of the historic farm management show that prior to 2010 the field comprised two separate areas (north 1.5 ha and south 5.6 ha) with contrasting management histories. The northern part had been managed as permanent grassland for at least 25 years, whereas the southern part has been ploughed three times in the last 25 years, most recently in September 2007 when it was re-seeded with a ryegrass/clover mixture following a previous winter barley crop. At the time of the study, both areas of the field had the same vegetation cover being classified under the National Vegetation Classification (NVC) category: “MG7 *Lolium perenne Poa trivialis* and related grasslands”. The southern region, however, had a higher clover content (*Trifolium repens*), less dense vegetation cover and approximately double the sward height compared to the northern part. These management histories are representative of normal management cycles of intensive grassland management (Bilotta et al., 2007).

### 2.2 Sample design, collection and analysis

To quantify spatial variability in δ^18^O_PO_4__, a sampling pattern was chosen with the view of a geostatistical analysis not only to δ^18^O_PO_4__, but to other soil variables, as presented in Peukert et al. (2012). In order to assess spatial variability across three different spatial scales, 78 soil samples were taken in total with samples at: (i) a broad scale (75 × 75 m grid); (ii) an intermediate scale (25 × 25 m grid); and (iii) a small scale (10 × 10 m grid). A hand-held GPS (Nomad Trimble, Sunnyvale, USA) was used to map and mark the sampling points.

All samples were collected in May 2011 to a soil depth of 7.5 cm and were oven dried at 105 °C for 24 h. Dried soils were then sieved through a 2 mm mesh. Total P (TP) was determined at an external laboratory (Natural Resource Management, Berkshire, U.K.) through digestion of the soils ground to 0.5 mm in aqua-regia followed by subsequent analysis on an ICP-AES. The δ^18^O_PO_4__ of the soil was determined on the 1 M HCl extractant described by Tamburini et al. (2010) but with a few modifications. Briefly, between 10 and 20 g dry soil was added to 100 ml 1 M HCl and shaken overnight. The supernatants were collected after separation from the residual solids by centrifugation and filtration. Phosphate concentrations were determined colourimetrically on an Aquachem 250 analyser using a molybdenum blue reaction (Murphy and Riley, 1962) after they were diluted by at least 1/10 to avoid acid interference with the molybdenum chemistry. The extracted PO_4_ is precipitated and dissolved as firstly ammonium phospho-molybdate and then magnesium ammonium phosphate before excess magnesium and chloride is removed through the addition of a cation resin and a small dose of silver nitrate crystals respectively. The resultant PO_4_ in solution is then converted to silver phosphate (Ag_3_PO_4_) though the addition of an Ag-ammine solution and subsequent adjustment of the pH to between 7 and 8 with 0.5 M HNO_3_ before incubation for two days at 50 °C in an oven.

Soil PO_4_ extracted using 1 M HCl (HCl_PO_4__) represents an integration of several potential PO_4_ pools (Zohar et al., 2010): (i) the most labile, water-extractable soil PO_4_, including intracellular microbial PO_4_ typically released through processes such as soil drying and re-wetting and cell lysis (ii) weakly adsorbed bicarbonate-extractable soil PO_4_, and (iii) strongly fixed, calcium and iron bound P. The weak acid extraction does not include the strongly adsorbed aluminium oxide bound PO_4_, which requires a NaOH-based extraction (Tiessen and Moir, 1993), nor does it include P in organic matter, the hydrolysis of which with 1 M HCl has been shown to be negligible during the development of the extraction protocol by Tamburini et al. (2010). To confirm that organic P forms were not being hydrolysed, duplicate soil samples were extracted using ^18^O-labeled and unlabelled 1 M HCl. If the extracted HCl_PO_4__ was to contain large amounts of hydrolised organic P or condensed PO_4_ species (e.g. polyphosphates, pyrophosphates, etc.) then the δ^18^O_PO_4__ of the sample duplicates would be markedly different. For all tested samples, the ^18^O of the sample extracted with unlabelled and ^18^O-labeled acid was no > 1%. The contribution of calcium-bound P is also considered to be negligible as both the soil parent material was neither igneous nor calcareous, and the soil pH is generally < 6. Therefore HCl_PO_4__ is assumed to extract PO_4_ from the same soil pool as is released by water, anion resins, and NaHCO_3_ extraction, namely extracellular labile PO_4_ and metabolic intracellular microbial PO_4_ and also some iron bound PO_4_. Using the 1 M HCl as an extractant enables far greater quantities PO_4_ to become available, quantities that are required to allow the successful precipitation of Ag_3_PO_4_ from small amount of soil.

Oxygen isotope analysis was carried out ETH Zurich on a Vario Pyro Cube (Elementar GmbH, Hanau, Germany) coupled in continuous flow to an Isoprime 100 isotopic ratio mass spectrometer IRMS (Isoprime, Manchester, UK). Triplicate samples of ~ 0.3 mg of Ag_3_PO_4_ were weighted into silver capsules together with a small amount of fine carbon black powder to promote the reaction between the Ag_3_PO_4_ and carbon to produce CO. The produced reaction gases are carried through a temperature-controlled chromatography column and ultimately to the IRMS. Calibration and corrections for instrumental drifts were done by repeated measurements of an internal standard (ACROS Ag_3_PO_4_ > 97.5%, δ^18^O = + 14.2‰; measured and calibrated at the University of Lausanne by T. Vennemann), and of benzoic acids IAEA 601 and IAEA 602 (+ 23.3‰ and + 71.4‰, respectively). The δ^18^O values are given in the standard ‰ notation with respect to VSMOW (Vienna Standard Mean Oceanic Water). Silver phosphate standards are routinely analyzed, and standard deviation on analytical replicates was better than 0.3‰.

### 2.3 Statistical analyses

Unfortunately, the described sampling design of Peukert et al. (2012) was flawed due to a mis-interpretation of the requirements needed for an efficient statistical analysis. The design resulted in: (i) large areas of gross under-sampling or voids and (ii) areas of preferential or clustered sampling due to the chosen sampling scheme. Both flaws also interact and compound each other. Given this, the direct application of many statistical methods is likely to be inefficient and sub-optimal, resulting in biased outputs (Diggle et al., 2010, Gelfand et al., 2012, Olea, 2007). As a consequence, all of this study's statistical analyses had to be conducted in a manner that would account for this sub-optimal sample design.

As a first step to mitigate against possible biasing effects, the following data pre-processing actions were taken: (1) the limits of the study area were set to lie significantly within the field boundary using a 10 m buffer around the all sample points that lie to the edge; (2) the data were declustered by removing observations that contribute the most to the preferential sampling; and (3) to complement Action 2, one-point declustering weights were found using the algorithm of Deutsch and Journel (1998), that enables the full data set to be used but where the clustered data are down-weighted (and so negate potential bias).

The statistical analyses for assessing the variation in δ^18^O_PO_4__ accorded to the following four linked stages: (i) a standard exploratory analysis; (ii) a variographic analysis to assess spatial dependence (e.g. Goovaerts, 1997); (iii) a geographically weighted (GW) correlation analysis (Fotheringham et al., 2002, Harris and Brunsdon, 2010) to investigate spatial heterogeneity in paired relationships; and (iv) a confirmatory regression analysis based on that observed in stages (i) to (iii), where δ^18^O_PO_4__ is taken as some function of HCl_PO_4__, elevation, slope, soil class and the historical north-south division. In addition to these analyses, that are centred on δ^18^O_PO_4__, complementary spatial analyses were conducted on TP and HCl_PO_4__ to provide useful context. All study hypothesis test results are reported at the 95% significance level; and all statistical analyses were implemented in R (http://www.r-project.org).

#### 2.3.1 Standard exploratory analysis

Conditional boxplots and conditional scatterplots were used to assess δ^18^O_PO_4__ distributions and relationships. This included evidence for multiple populations for δ^18^O_PO_4__, according to the historical north-south field division or to soil class; and whether or not the relationship for δ^18^O_PO_4__ with HCl_PO_4__, with elevation and with slope; changed according to the north-south division. Multiple linear regression (MLR) fits were conducted using a step-wise, ordinary least squares (OLS) estimation that finds the best predictor variable subset according to the Akaike Information Criterion (AIC). Considering the data is spatial, the OLS fits were viewed as exploratory; definitive spatial regression fits are described in Section 2.3.2, below. Hypothesis tests relating to this stage, follow the usual parametric approach via *t*-tests.

#### 2.3.2 Variographic analysis

To assess spatial dependence in δ^18^O_PO_4__, we limited our investigations to: (i) raw data variograms; (ii) residual data variograms from the OLS MLR trend fits of Section 2.3.1; (iii) (outlier-resistant) robust variograms (Hawkins and Cressie, 1984); (iv) within-class variograms (e.g. Goovaerts, 1997); (v) local variograms (where (iv) and (v) accord to the historical field division); (vi) normal-scores transformed data variograms; and (vii) cross-variograms with HCl_PO_4__. Lags for all empirical variograms were chosen to reflect the study's sample design; and only omni-directional variograms were found. To counter any biasing effects on variogram estimation caused by the sample design, a two-point declustering algorithm was also used to provide weighted variograms (Richmond, 2002). For variograms that displayed spatial structure, they were modelled using a weighted least squares (WLS) approach specified with an exponential variogram model-type.

For investigation (ii), an OLS MLR fit and its corresponding WLS residual variogram model fit are sub-optimal (but often informative in an explorative context). As such (and when required), both sets of parameters (i.e. those for the MLR and those for the variogram) were optimally re-estimated using restricted maximum likelihood (REML) (e.g. Schabenberger and Gotway, 2004). REML is also useful in that it can similarly account for variogram bias due to the sample design, as can the two-point declustering algorithm, above (Marchant et al., 2013).

#### 2.3.3 Geographically weighted correlations

Spatial heterogeneity in δ^18^O_PO_4__ relationships was investigated via the mapping of GW correlations. These localised correlations are found at the sample sites of the study area, where they are calculated in the same manner as their standard (global) counterpart, but only use data that are spatially nearby to the sample locations. Nearby data are spatially weighted (via a kernel weighting function), so that the closest data points are given more influence in the local statistic. Geographically weighted correlations form one of a suite of GW methods (Fotheringham et al., 2002, Lu et al., 2014, Gollini et al., 2015), another of which, GW regression (GWR) is described below.

A GW method can be viewed as a moving window weighting technique, where the size of the window over which any localised statistic/model might apply is controlled by the kernel's bandwidth. Commonly, this exploration of spatial heterogeneity involves: (i) a carefully judged choice of bandwidth; (ii) a test for significance of the observed heterogeneity; together with (iii) a mapping of the outputs. For this study, we specified the GW correlations using an adaptive Gaussian kernel. In the absence of an objective procedure for bandwidth selection, we experimented with three user-specified bandwidths of 30%, 40% and 50%. Hypothesis tests for this stage, followed a randomisation approach where the true local correlation is compared to that found from 999 random permutations of the data (e.g. Fotheringham et al., 2002).

#### 2.3.4 Regression analysis

The following three regressions were considered, where δ^18^O_PO_4__ is the response variable: (i) MLR; (ii) MLR with spatially-autocorrelated error; and (iii) GWR (Brunsdon et al., 1996). The first two regressions assume data relationships are globally-fixed, while the third regression allows data relationships to locally-vary. The parameters of the two MLR models can be estimated by OLS and REML, respectively. Similar to GW correlations, GWR enables the exploration of data relationships, where localised regressions are fitted using spatially weighted data and the resultant regression coefficients are mapped. GWR parameters are estimated using a WLS procedure, where we again specify an adaptive Gaussian kernel weighting scheme. For GWR, an objective function exists for bandwidth selection; and here we use an automatic AIC procedure (Fotheringham et al., 2002). To compare the fit of chosen regressions, AIC and R^2^ values are reported.

Hypothesis tests for significant regression coefficient heterogeneity follow both: (i) a randomisation approach (Brunsdon et al., 1998) and (ii) a parametric bootstrap approach (Harris et al., 2015). For the former, GWR is applied to 999 random permulations of the data and the variance of a given coefficient is found. The actual variance of the same coefficient is then included in the ranked distribution of variances, where its position (i.e. its *p*-value) relates to whether there is significant spatial variation in this coefficient.

For the latter, bootstrap samples of the response variable are found for a null (fixed coefficient) model (e.g. MLR), where the simulations are based on the estimated parameters of the null model fit to the sample data. The predictor variables are not considered random and are the same as the sample data. A test statistic *q* that measures the spatial variability in the GWR coefficients is then used to test against the null hypothesis. Thus a GWR model is fitted to each bootstrap sample and a *q*-statistic is found for each regression coefficient. As the null model is a random process, even when coefficients do not vary spatially, one would not expect the GWR coefficients to be identical in different locations. The bootstrap analysis determines how much coefficient variability one might expect to encounter due to random variation in a model, and to compare the level of variability in the observed data set, against this.

The bootstrap tests are run with the number of simulations set at 999. For each regression coefficient, the 95% points of the bootstrap samples are computed, and significance levels are found for upper single-tailed hypothesis tests. In addition, at every sample (i.e regression point) location, the localised set of regression coefficients are tested for significant difference to their corresponding fixed coefficient estimates. Here at each sample location, a bootstrap sample of pseudo *t*-values (e.g. Harris et al., 2010) are found for each coefficient, enabing a bootstrap *p*-value to be found accordingly. Mapping these *p*-values identifies where local coefficients significantly differ to their fixed (or global) coefficient counterpart.

## 3 Results

### 3.1 Distributions of TP, HCl_PO_4__ and δ^18^O_PO_4__ with the raw clustered data

The TP of the soil across Great Field ranged from 736 to 1952 mg P kg^− 1^, while the HCl_PO_4__ ranged from 93 to 821 mg P kg^− 1^ extracting between 12 and 48% of the TP present in the sample. As would be expected there was a strong positive correlation between TP and HCl_PO_4__ (*r* = 0.91), where the proportion of TP as HCl_PO_4__ was found to increase with increasing soil TP content. The δ^18^O_PO_4__ of the HCl_PO_4__ ranged from 17.0 to 21.6‰ with a mean of 18.8‰ (± 0.8).

**Fig. 1:**
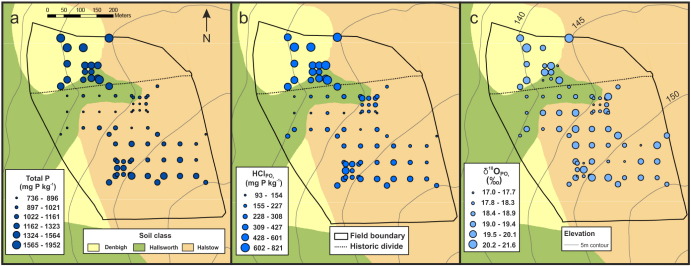
The spatial distribution of (a) TP, (b) HCl_PO_4__, and (c) δ^18^O_PO_4__ of the HCl_PO_4__ within ‘Great Field’ on the North Wyke Farm Platform.

The spatial distributions of TP, HCl_PO_4__ and δ^18^O_PO_4__ are presented in Fig. 1a–c. The historic field divide appears an important discriminating variable in terms of both TP and HCl_PO_4__ with higher values to the north of the divide. Soil class also appears a useful discriminator of TP and HCl_PO_4__. The study field slopes downwards from its south-east corner to broadly where the historical divide starts to the west; and similarly slopes downwards from the north to the same point. Thus the field is broadly concave in shape, and this also appears to influence the distribution of TP and HCl_PO_4__. Conversely, there does not appear to be any spatial trend in δ^18^O_PO_4__ or environmental factors that influence its variability. Note that all data descriptions in this section are naïve given that any bias due to the sample design, are not (as yet) considered.

### 3.2 Actions taken to address sub-optimal sample design

**Fig. 2:**
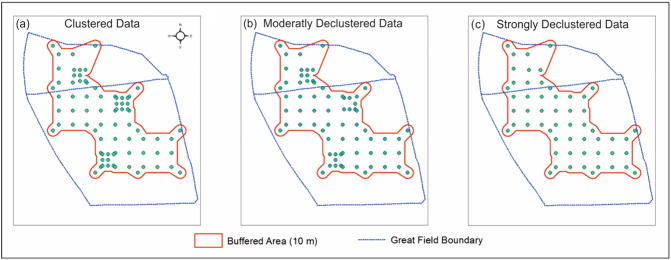
The distribution of: (a) clustered data, (b) moderately declustered data and (c) strongly declustered data.

As a first action to address potential sub-optimality (Action 1, from Section 2.3), a 10 m buffer was used around the data to limit all analyses to only a sub-region within the study field (Fig. 2). For Action 2 the data were both moderately and strongly declustered by removing 7 and 22 observations, respectively through expert judgement. Actions 1 and 2 are depicted in Fig. 2. Both declustered data sets simply lessen the impact of the three main areas of clustering, where data have been manually removed according to their location (and not by their measurements). The main drawback to the use of declustered data is the reduced information, which is already limited to 78 locations.

Next, we assessed for bias in the (global) means of TP, HCl_PO_4__ and δ^18^O_PO_4__, according to the three different data sets depicted in Fig. 2. An alternative set of weighted means were also calculated using weights found from a cell-declustering to a 25 m grid cell (a natural choice given the design); which is Action 3 from before. Results are presented in Table 1, where the clustered data tends to: (i) slightly under-estimate the mean for TP; (ii) slightly over-estimate it for HCl_PO_4__; and (iii) very slightly under-estimate it for δ^18^O_PO_4__. Results suggest that continuing with the clustered data is reasonable, with a proviso that only models that cater for possible bias are applied. Weighted correlations and weighted regressions (WLS MLR) can also be found using the clustered data, where we assume that the cell-declustering weights found for an unbiased global mean (for δ^18^O_PO_4__ in Table 1), are also suitable to down-weight data relationships in areas of clustering. It is also prudent to calibrate models with the strongly declustered data; and their outputs compared with models calibrated with the clustered data.

### 3.3 Complementary analyses for TP and HCl_PO_4__

**Fig. 3:**
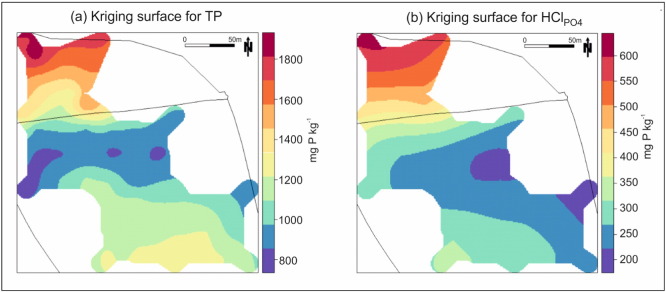
The spatial distribution of (a) TP and (b) HCl_PO_4__; each found using a KED model.

Although our focus is the spatial analysis of δ^18^O_PO_4__, it is useful to provide an insight into how TP and HCl_PO_4__ vary spatially. This provides context to the analysis of δ^18^O_PO_4__, especially as we choose to investigate its relationship to HCl_PO_4__ in subsequent sections. As opposed to TP, HCl_PO_4__ more directly relates to our understanding of δ^18^O_PO_4__. Thus in Fig. 3, prediction surfaces are given for TP and HCl_PO_4__; both of which were constructed using a kriging with external drift (KED) model, with all parameters estimated optimally via REML, and a global neighbourhood specified. For TP, its KED trend component was informed by elevation, slope and the north/south division; whereas for HCl_PO_4__ its trend was informed by elevation, slope, soil class and the north/south division. The R^2^ values for the trend fits (i.e. via OLS) were strong at 0.69, in both cases.

Observe that for the trend component of each KED model, TP and HCl_PO_4__ were not used to help predict each other, as this would not inform predictions on a grid (δ^18^O_PO_4__ was similarly not used in this respect). Variography for both KED models was reasonably well-behaved, with a clear single structure depicting spatial dependence up to distances of around 100 m. As both variables are highly correlated, their parameterisation and surfaces are broadly similar. The highest TP and HCl_PO_4__ values clearly lie to the north of the historical division, while the lowest values lie in a broad swathe south and east of the historical division.

All analyses in this section were conducted on the clustered data, but with consideration to potential bias. Observe that the clustered data can be used when kriging (i.e. after its parameterisation), as it inherently accounts for such configurations via its information, screening and relay effects (Chilès and Delfiner, 1999).

### 3.4 Exploratory analysis for δ^18^O_PO_4__

**Fig. 4:**
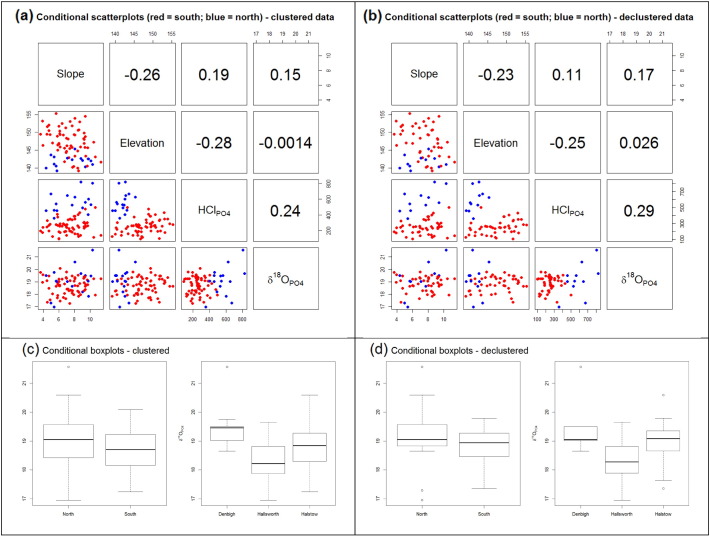
Scatterplots and correlation coefficients for the (a) clustered and (b) strongly declustered data, respectively. These display the pairwise relationships between δ^18^O_PO_4__, HCl_PO_4__, elevation and slope. Points coloured red and blue relate to samples to the south and north of the historic field division, respectively. Conditional boxplots for δ^18^O_PO_4__, with respect to the historic field division or the three soil classes for (c) clustered and (d) strongly declustered data, respectively.

Using both the clustered and strongly declustered data, the relationship matrices for δ^18^O_PO_4__, HCl_PO_4__, elevation and slope are presented in Fig. 4a & b. Correlation coefficents are given for the complete data sets, while scatterplots depict relationships that are conditional to the historic field division. The latter of which, provides a first insight into possible local relationships. Weighted correlations using the cell-declustering weights are also given in Table 2. By focusing only on those relationships with δ^18^O_PO_4__, the strongest relationship is with HCl_PO_4__, but this is weak with a correlation of only 0.30 (for the weighted correlation). Clearly, δ^18^O_PO_4__ poorly correlates with elevation and with slope. It appears that data relationships may be conditional to the historic field division; and in particular, the relationship of δ^18^O_PO_4__ with HCl_PO_4__.

Conditional boxplots are used in Fig. 4c & d to relate the distribution of δ^18^O_PO_4__ to both the historic field division and to soil class; again using both clustered and strongly declustered data. Marginally higher δ^18^O_PO_4__ values are generally found in the northern part of the field, but in general, discrimination is poor. Similarly, the three soil classes do not appear to be a strong discriminator of δ^18^O_PO_4__. In general, there is little to choose between the clustered and declustered data analyses. Thus the sample design does not appear to strongly bias δ^18^O_PO_4__ in this respect. Regardless of any biasing effects, all relationships for δ^18^O_PO_4__ are weak or indistinct. Locally however, this may not be the case, and we investigate this further in 3.6, 3.7.

Table 3, Table 4 present the results from a series of MLR fits to the clustered and strongly declustered data, with δ^18^O_PO_4__ as the response. Table 3 reports the results using all predictors (HCl_PO_4__, elevation, slope, soil class and north/south division), while Table 4 reports the results using predictor subsets chosen via step-wise AIC. In both cases, WLS MLR fits are also reported using the cell-decustering weights from before. Clearly, all MLR fits are poor, where the highest R^2^ is only 0.35. There is also no consistency in the make-up of the predictor subset or in the significance of those variables chosen (note that significance tests are naïve in that any spatial dependence in the data is not as yet considered). For the former, this is not surprising given that reductions in AIC are consistently small. Unlike the previous analyses, it now appears that the sample design is detrimental to fitting regressions (as the poorest R^2^ values result when directly using the clustered data). None of the predictor variables appear particularly worthy predictors of δ^18^O_PO_4__ in this global sense, but it is unclear which, if any varables, could be safely removed, before we proceed to more detailed analyses. Given these analyses, *study hypothesis* (*A*) *is accepted*.

### 3.5 Variographic analysis for δ^18^O_PO_4__

**Fig. 5:**
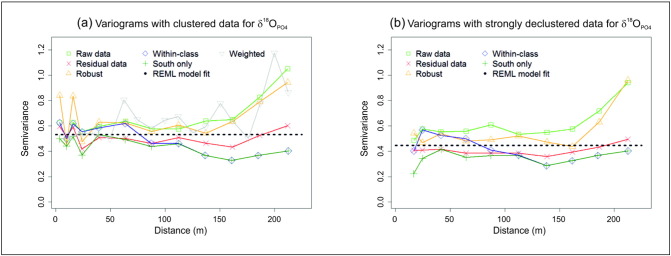
Variograms for δ^18^O_PO_4__ using (a) clustered data and (b) strongly declustered data.

We next investigate for spatial dependence in δ^18^O_PO_4__ using raw data, residual data, robust, local and within-class empirical variograms. Again, we compare results using the clustered and strongly declustered data. For the residual variograms, OLS MLR trend fits with all predictor variables are used. We also provide a weighted variogram to the clustered data and REML model fits to both data sets using all available predictors to inform the trend. All variograms are given in Fig. 5, where only very weak evidence for spatial dependence is found and the δ^18^O_PO_4__ data is essentially random. This is endorsed by the REML results, where the AIC for the spatial model was 4 units higher than the corresponding non-spatial model for the both data sets. Unsurprisingly, as spatial dependence was absent in δ^18^O_PO_4__, cross-dependence with HCl_PO_4__ was also absent (even though spatial dependence is present in HCl_PO_4__, from Section 3.3). Given these results, it is unnecessary to formally test for spatial dependence in δ^18^O_PO_4__ and *study hypothesis* (*B*) *is accepted*.

From Fig. 5, the effects of data clustering on the variograms are quite evident, where semi-variances at the lower lags of the clustered data variograms are commonly heightened, resulting in very poor small-scale structure. Conversely, most of the strongly declustered data variograms depict small-scale structure, but no longer have any semivariance values at the lowest lag distances. The differences between the raw data variograms (using complete data sets) and the south data variograms indicate that δ^18^O_PO_4__ has a higher variance in the north than that found in the south. The behaviour of the within-class variograms somewhat endorses this (noting that they mimic the south variograms at higher lags as no such semivariance pairs exist in the north). North data variograms were not found as there were too few data points to compute a valid variogram. All residual variograms clearly depict pure nugget effects, as do the REML models. Finally, normal-scores variograms were also found, but did not help in structure identification.

### 3.6 Local relationships for δ^18^O_PO_4__

Given that spatial autocorrelation effects are absent for the δ^18^O_PO_4__ process, out next objective is to assess for any spatial heterogenic effects with respect to the relationships between δ^18^O_PO_4__ with HCl_PO_4__, elevation, and slope. Furthermore, if such heterogeneities exist, do they depend on the historical field divison or alternatively, the under-lying soil class?

**Fig. 6:**
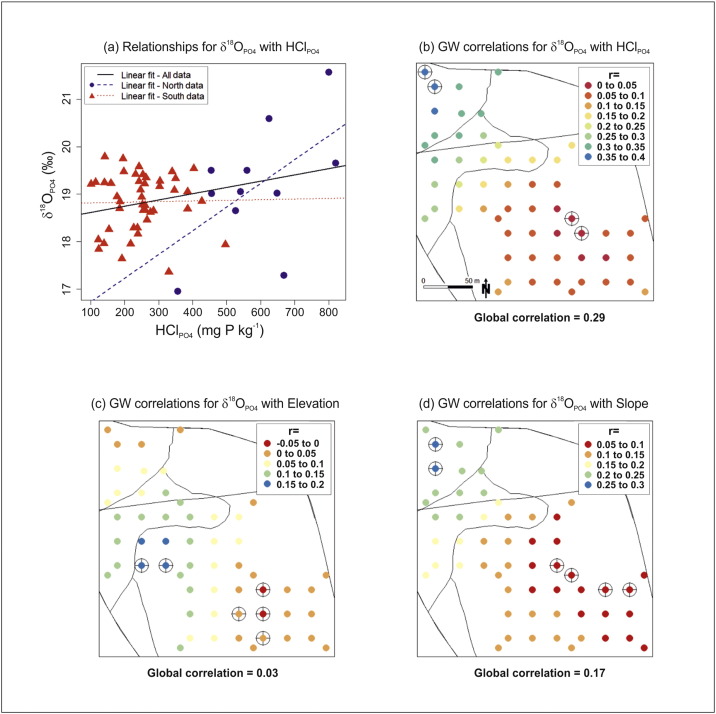
(a) Strongly declustered data conditional scatterplot for δ^18^O_PO_4__ with HCl_PO_4__. Points are separated on their location in the field relative to the historic field division. Lines of best fit, using: all of the data, north data only, and south data only are shown. Geographically weighted correlations for δ^18^O_PO_4__ with (b) HCl_PO_4__, (c) elevation, and (d) slope - at the strongly declustered data locations. Associated randomisation test results are circled, indicating locations of unusual correlation. Maps are given with the historical field division and the three soil class divisions.

Evidence for such heterogeneities have already been observed in Fig. 4, where the relationship between δ^18^O_PO_4__ and HCl_PO_4__ appears conditional to the historic field division. In order to explore this particular relationship further, the same conditional scatterplot is given in Fig. 6a, using the strongly declustered data. Here we calculate correlations for the northern and southern parts of the field. Given that spatial autocorrelation effects are absent (both globally and within each partition), and that the declustered data is used, we can report the significance of these correlations (using standard *t*-tests with correlations) with relative assurance. Here the global correlation of 0.29 between HCl_PO_4__ and δ^18^O_PO_4__ is significantly different to a zero correlation, whereas the local correlation in the south is very weak at 0.02 and not significant. In the north, the local correlation was relatively strong at 0.55, but given that it is found using only eleven data points, this correlation was also insignificant. Observe that we have shown the data with their linear fits, where the intercept and slope vary locally. This concept of spatially-varying regression coefficients is re-visited in Section 3.7. Note that as we are investigating locally, it is assumed that an unbiased analysis will result by only using the strongly declustered data (see Section 4).

To further our investigations of relationship heterogeneity, Fig. 6b–d presents GW correlation maps for δ^18^O_PO_4__ with HCl_PO_4__, with elevation, and with slope; each specified with a bandwidth of 40%. Co-variability for δ^18^O_PO_4__ with HCl_PO_4__ tends to be stronger in the northern part of the field (Fig. 6b), and these findings are endorsed by the associated randomisation tests that indicate areas of unusually strong correlation, as well as unusually weak correlation in the south. The distribution of GW correlations between HCl_PO_4__ and δ^18^O_PO_4__ largely confirm that found in Fig. 6a, but with more detail. As a continuous (Gaussian) weighting scheme is specified, all GW correlations are actually informed by all 58 observations of the strongly declustered data (a bandwidth of 40% entails that the nearest 22 observations exert the greatest influence on each localised correlation). This weighting specification is considered crucial to a GW analysis to such a relatively small data set. Thus the GW correlations in the north and the south of the field are better informed than the two partitioned correlations from before. From Fig. 6c–d, correlations for δ^18^O_PO_4__ with elevation, and with slope, only marginally vary across the field. It is possible that all such heterogeneities are dependent on the field division (and therefore different managements) or the under-lying soil class.

### 3.7 Regression analysis for δ^18^O_PO_4__

Given the analyses of the preceeding sections, only MLR (estimated using OLS) and GWR models need to be considered. Extending MLR to account for spatial autocorrelation in the residual term is unnecessary given the residual data variograms and REML results of Section 3.5. Models are applied, using the strongly declustered data only and all predictor variables are used. Thus the MLR model is the same as that given in Table 3. The bandwidth for the GWR model is optimally found at 88%, indicating a shallow weighting and suggesting weak relationship heterogeneity. The model fit results for MLR and GWR give AIC values of 125.4 and 124.6, respectively; together with R^2^ values of 0.35 and 0.38, respectively. Clearly, there is little to be gained from applying GWR in preference to MLR. Table 5 tests for coefficient heterogeneity using: (i) the parametric bootstrap approach with MLR as the null model and (ii) the randomisation approach. Clearly, and as expected, there is no significant evidence for coefficient (i.e. relationship) heterogeneity in this data, as all *p*-values are > 0.05. Given these test results, *study hypothesis* (*C*) *is accepted*.

**Fig. 7:**
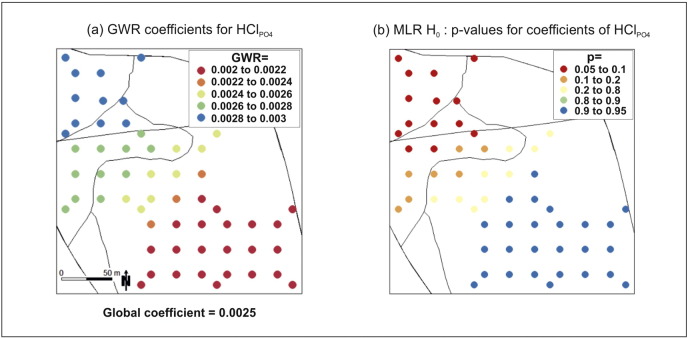
The (a) GWR coefficients for HCl_PO_4__ with a bandwidth of 88% and (b) the associated results from parametric bootstrap test.

The localised regression coefficients from GWR associated with HCl_PO_4__ are mapped in Fig. 7a, where it appears that the relationship for δ^18^O_PO_4__ with HCl_PO_4__ varies spatially. Furthermore, this relationship appears to depend on the historical field divison and/or the under-lying soil class. There is also a north-west to south-east trend in the coefficients, reflecting the shallow weighting function that was specified. In Fig. 7b, the bootstrap *p*-values for the local sets of pseudo *t*-values indicate where the coefficients associated with HCl_PO_4__ are significantly smaller (to the north) and significantly larger (to the south) than the global value. However from the map legend, this only relates to a 90% significance level and not the stated study significance level of 95%. Thus, the local coefficients are not significantly different to their global counterpart, and as such, *study hypothesis* (*D*) *is accepted.* Observe that given the results of Table 5, we do not further investigate the localised regression coefficients for the intercept or the other predictor variables.

## 4 Discussion

### 4.1 Variability of HCl_PO_4__ and TP

Analyses for HCl_PO_4__ and TP indicate that both variables are spatially autocorrelated; where elevation, slope, soil class and the historic field divide can influence this variability. However the historic field divide is considered the most important driver, with higher values to the north of the divide than to the south. This is not unexpected given the management differences between the two sectors and is because the northern part of the field has not been ploughed for a significant time, while the southern part had been ploughed and most recently in 2007. The surface enrichment of P is normal in agricultural soils, especially grasslands which may not be ploughed frequently, and reflects the accumulation of P from inorganic fertilizers and manures over time. The decrease in P with depth is often marked over a few cm with the bulk of the soil profile considerably lower in P. Where ploughing has occurred, such profiles are destroyed within the plough zone and the high P surface soil diluted or replaced with lower P soil brought up from depth (e.g. Haygarth et al., 1998a, Haygarth et al., 1998b, Watson and Matthews, 2008). The amounts of the HCl_PO_4__ indicate that > 50% of P in the soil is in 1 M HCl recalcitrant forms such as organic P or aluminium oxides. Given the negligible contribution of bedrock apatite PO_4_ it is assumed that the bulk of the HCl_PO_4__ is comprised of PO_4_ adsorbed to soil particles and microbial PO_4_. The soils in this region have been shown to have a high microbial biomass P content, which in turn has been shown to be released upon drying and rewetting through cell lysis (Blackwell et al., 2013). The amounts of HCl_PO_4__ measured are very similar to the levels of microbial P reported by Blackwell et al. (2013) and therefore we assume that HCl_PO_4__ in these soils represents, in large part, adsorbed soil PO_4_ and intracellular microbial PO_4_, the latter normally exceeding the former (Blackwell et al., 2010).

### 4.2 Variability of δ^18^O_PO_4__

In order to discuss δ^18^O_PO_4__ values observed, we need to estimate the theoretical equilibrium δ^18^O_PO_4__ value that might be expected for PO_4_ in equilibrium with soil water which is believed to be mediated by the ubiquitous intracellular enzyme pyrophosphatase (Blake et al., 2005). This causes the exchange of PO_4_ oxygen with the oxygen in H_2_O and results in a temperature dependent relationship initially described by Longinelli and Nuti (1973). However recent work by Chang and Blake (2015) has developed a refined, rigorous and controlled laboratory calibration of the temperature-dependence of equilibrium PO_4_ and water, catalyzed by pyrophosphatase, over a typical environmental temperatures (3–37 °C):

Eδ^18^O_PO_4__ = − 0.18 T + 26.3 + δ^18^O_H_2_O_where Eδ^18^O_PO_4__ is the stable oxygen isotope ratio of PO_4_ at equilibrium in ‰, T is the temperature in degrees Celsius and δ^18^O_H_2_O_ is the stable oxygen isotope ratio of H_2_O in ‰. The intracellular phosphate, already at equilibrium, is released to the soil after cell lysis.

Although measurements of soil water δ^18^O_H_2_O_ were not directly made it has been suggested that soil water at the location is very similar δ^18^O_H_2_O_ to ground water, in that it is an integrated value of many rainfall events (Granger et al., 2010). As such the δ^18^O_H_2_O_ should be similar to that of the global meteoric water line for this area with δ^18^O_H_2_O_ ranging between − 5.5 to − 6.0‰ (Darling et al., 2003). The average soil temperature measured at 10 cm depth nearby was 13 °C for the month of May and this would give estimated vales of δ^18^O_PO_4__ at equilibrium with soil water of between 18.0 and 18.5‰. However given the uncertainties in these assumptions it is wise to examine a window of potential soil δ^18^O_PO_4__ and temperature values to understand better the measured δ^18^O_PO_4__ data compared to the theoretical equilibrium δ^18^O_PO_4__ values. For example, it might be expected that the integrated soil temperature of the soil profile from surface to 7.5 cm depth is slightly warmer than that measured at 10 cm. Further, although ground water is an analogue for soil water, the variability of soil water δ^18^O_H_2_O_ is more pronounced and subject to strong influence from individual rainfall events and also from enrichment in ^18^O through evapotranspiration, especially in the surface of the soil (e.g. Hsieh et al., 1998, Treydte et al., 2014). Therefore if it is assumed that the integrated 7.5 cm profile soil temperature ranges between 13 and 15 °C, and that the δ^18^O_H_2_O_ is enriched by up to 4‰ compared to groundwater, then the theoretical δ^18^O_PO_4__ equilibrium values range between 18.0 and 23.0‰. This range of values better matches the δ^18^O_PO_4__ of the soil and suggests that the HCl_PO_4__ within the soil is at or around equilibrium. This finding is in common with other researchers. Angert et al. (2012) found that across a Mediterranean bed rock and rainfall gradient, both soil resin extractable PO_4_ and HCl_PO_4__ were broadly in equilibrium with soil water and proposed, amongst other reasons, that a flux of intracellular equilibrated PO_4_ from the soil microbial biomass may be causing this. Tamburini et al. (2012) also conclude that in young and developing alpine soils, regardless of the contribution of PO_4_ from minerals or vegetation, or from the activity of extracellular enzymes, microbial biomass was the main controller of the δ^18^O_PO_4__, keeping it in equilibrium with soil water. In both these examples however the soils were generally low in P, and where P is limiting it is expected that microbial cycling would be rapid. Within this current study soil P should not be limiting and it is therefore interesting that δ^18^O_PO_4__ would still appear to be relatively rapidly cycled and have no obvious trace of any P source signatures. One explanation for this, would be if the predominant P sources had δ^18^O_PO_4__ signatures similar to that of the equilibrium δ^18^O_PO_4__ value. The δ^18^O_PO_4__ of inorganic fertilizers have been reported to show a very wide range of values from 14.8 to 27.0‰ while virtually no δ^18^O_PO_4__ values have been published for animal excreta (Young et al., 2009) or for stored and managed animal wastes, however, water-extractable PO_4_ from dairy farm slurries in this area have been found to range from 12.0 and 15.0‰ (Granger, unpublished data).

Analysis results for δ^18^O_PO_4__ relationships within the data are enigmatic. Considering the field as a whole, all relationships for δ^18^O_PO_4__ are weak or indistinct. HCl_PO_4__, elevation, slope, soil class and the historic field divide are not particularly good drivers of δ^18^O_PO_4__ variability; and δ^18^O_PO_4__ is not spatially correlated with itself. For within-field relationships between δ^18^O_PO_4__ and HCl_PO_4__, elevation and slope; only the local relationship for δ^18^O_PO_4__ with HCl_PO_4__ appears to have any value, showing a positive relationship north of the historic field divide which does not exist to the south. However, such localised relationships are not significantly different to that found for the field as a whole. Thus in essence, δ^18^O_PO_4__ is not only randomly distributed with respect to itself, but also to the particular environmental factors that this study has considered.

If it had been found that the positive relationship between δ^18^O_PO_4__ with HCl_PO_4__ was significant in the northern part of the field, which this study can only allude to; this may imply that the process of ploughing, which has destroyed the soil P profile in the south, has also decoupled any link between HCl_PO_4__ and δ^18^O_PO_4__ that is present in the north. The scenario described by the data seems to suggest that, in the north, elevated HCl_PO_4__ occurs as ‘hotspots’ with associated higher δ^18^O_PO_4__, and that as the HCl_PO_4__ of these hotspots declines so the δ^18^O_PO_4__ becomes lower. Such P hotspots have been described previously under cattle grazing regimes which not only leads to an increasing the TP in the surface of the soil but also to a heterogeneous distribution of TP ‘hotspots’ as a result of livestock excreta (Page et al., 2005, Penn et al., 2007). However, the available data on δ^18^O_PO_4__ in excreta, although limited, indicates that it is not elevated. If however metabolic PO_4_ released from leaf litter has an elevated δ^18^O_PO_4__ (Pfahler et al., 2013) this might explain the difference between the north and south of the field. In the south, ploughing has buried and mixed soil organic matter, which in the north has had time to accumulate. Potentially, this accumulation may not be homogenous and may still be affected by the hotspots of excreta described previously or it might just reflect a heterogeneous accumulation of non-excretal plant material within the soil profile. However if this is the cause of the relationship between δ^18^O_PO_4__ and HCl_PO_4__ it is not reflected in the soil total carbon content which shows no relationship with δ^18^O_PO_4__.

### 4.3 Controling variables

Throughout this study, we have not attempted to de-couple the effects of the historic field divide from the underlying soil class, in how these categorical variables influence variation and co-variation in HCl_PO_4__, TP and δ^18^O_PO_4__. It is clear from Fig. 1 that both categorical variables act as similar discriminators, as data only coincides with the Denbigh class in the northern part of the field, while the Halstow class primarily relates to data in the south. Such similarity in discriminating power is then clearly evident in the subsequent analyses. Given that sample size is small and many results are null, it was considered appropriate to maintain the controlling interaction between these categorical variables in all regression fits. However, as the distinction between the three soil classes is considered minimal in practise, any HCl_PO_4_,_ TP or δ^18^O_PO_4__ difference between the soil classes is considered more likely a reflection of the historic field division rather than a characteristic of the soil classes themselves.

### 4.4 Analysis limitations

Although the statistical analyses for δ^18^O_PO_4__ point to a series of null results, it should be borne in mind that the study data is limited in size and that the sampling configuration may miss the key scales of spatial dependence and co-dependence in δ^18^O_PO_4__. In this respect, future δ^18^O_PO_4__ (and HCl_PO_4__) studies should consider sampling on a finer grid with increased sampling, with nested sampling strategies (e.g. Atteia et al., 1994, Corstanje et al., 2008). Increased sampling should also guard against results simply reflecting sampling variation rather than the true properties of the spatial process.

Issues of sample design necessitated the use of the strongly declustered data for all localised analysis; and the associated loss of information. The only way to have proceeded with the clustered data would have required the calculation of localised sets of de-clustering weights, as it was not viable to use the globally-found ones of Section 3.2. As this would have presented a challenge for any partitioned analysis, and more so for any GW analyses (where the nature of the clustering bias would locally-vary according to the kernel and its bandwidth), the use of localised de-clustering weights was not considered.

Given the poor model fits throughout, the small sample size and the known issues for GWR with respect to local predictor variable collinearity (Páez et al., 2011), the results of Section 3.7 are viewed with caution. Although here, the GWR fit was assessed for adverse collinearity effects following the procedures outlined in Gollini et al. (2015), and although collinearity was present, its effects were not considered serious. Further work could extend the GWR analyses in this respect, where in addition to the fit of a penalised form of GWR, a mixed GWR (Brunsdon et al., 1999) could be considered where the categorical predictors are globally fixed, while the other predictors are allowed to locally-vary.

It is also worth noting that variation in δ^18^O_PO_4__ may be driven by environmental covariates not considered in this study, as such, future studies should consider identifying these missing covariates. Measurement error in δ^18^O_PO_4__ could also be mitigating factor in this study's null results and could be incorporated in future work, noting that the δ^18^O_PO_4__ values of this study represent an average of a three replicates.

## 5 Conclusions

This study has shown that, despite differences in elevation, slope, the under lying soil class and management, the δ^18^O_PO_4__ has no relationship with these field variables even though the HCl_PO_4__ can have. The soil δ^18^O_PO_4__ values within the field were found to be within the range predicted for that has been microbially cycled and is at equilibrium with δ^18^O_H_2_O_. However given the lack of information of PO_4_ source δ^18^O_PO_4__ signatures, it is not clear whether this is due to the rapid microbial cycling of PO_4_ or because PO_4_ sources have a similar δ^18^O_PO_4__ to that of the equilibrium value. Although no relationships were found between the δ^18^O_PO_4__, and itself (with respect to spatial autocorrelation) and the field variables investigated, there was a suggestion of a positive correlation between δ^18^O_PO_4__ and HCl_PO_4__ in the northern unploughed, field sector which was not present in the southern ploughed part of the field. While no conclusive evidence has been found to explain this, it has been suggested that this might be related to the metabolic PO_4_ within plant material heterogeneously accumulating in the soil surface in the unploughed part of the field.

This study provides an important advance to understanding spatial dependencies and spatial relationships in phosphate stable oxygen isotopes within a temperate agricultural soil. Some variability information was as hypothesised while intriguingly others were not. Given this, further work is ear-marked to learn and benefit from this study's results, via the implementation of a coherent multivariate sampling design, to a different field, with a known long-term management history.

## Figures and Tables

**Table 1:** Global means of clustered and declustered data sets.

Variable	Clustered data	Moderately declustered data	Strongly declustered data	Clustered data with weights
TP	1134.82	1146.49	1151.94	1145.61
HCl_PO_4__	317.77	322.37	314.73	311.79
δ^18^O_PO_4__	18.75	18.81	18.89	18.62

**Table 2:** Weighted correlations using the cell-declustering weights.

	Slope	Elevation	HCl_PO_4__	δ^18^O_PO_4__
Slope	1	− 0.30	0.14	0.19
Elevation	–	1	− 0.26	− 0.019
HCl_PO_4__	–	–	1	0.30
δ^18^O_PO_4__	–	–	–	1

**Table 3:** MLR fits using the full predictor variable set.

Data set	Estimation	R^2^	Predictor set	Significant predictors
Clustered	OLS	0.23	All predictors	Intercept, Hallsworth soil class
Clustered	WLS	0.31	All predictors	Intercept, Hallsworth soil class
Strongly declustered	OLS	0.35	All predictors	Intercept, HCl_PO_4__, Hallsworth soil class

**Table 4:** MLR fits using predictor variable subsets chosen by the step-wise AIC procedure.

Data set	Estimation	R^2^	AIC reduction	Predictor subset	Significant predictors
Clustered	OLS	0.22	4.6	Slope, soil class	Intercept, slope, soil class
Clustered	WLS	0.31	3.3	HCl_PO_4__, slope, soil class	Intercept, slope, Hallsworth soil class
Strongly declustered	OLS	0.34	1.2	HCl_PO_4__, elevation, north/south division, soil class	Intercept, HCl_PO_4__, elevation, Hallsworth soil class

**Table 5:** Bootstrap test *q*-statistics, associated *p*-values and randomisation test *p*-values.

	Intercept	HCl_PO_4__	Elevation	Slope	N/S division	Hallsworth soil class	Halstow soil class
Actual	0.726	0.000	0.006	0.006	0.091	0.026	0.059
MLR 95%	2.475	0.001	0.017	0.026	0.242	0.078	0.128
MLR *p-*value	0.266	0.140	0.220	0.400	0.208	0.193	0.158
Randomisation test *p*-value	0.275	0.096	0.200	0.575	0.443	0.900	0.584
